# Supplementary material for: Sex Differences and Predictors of In-Hospital Mortality among Patients with COVID-19: Results from the ANCOHVID Multicentre Study
Source: Int J Environ Res Public Health. 2021 Aug 26;18(17):9018. doi: 10.3390/ijerph18179018 (PMC8431708; doi:10.3390/ijerph18179018)
Supplement: Supplementary file 1 [file ijerph-18-09018-s001.zip › ijerph-1304189-supplementary2.pdf]

International Journal of
*Environmental Research and Public Health*

MDPI

**Table S1.** STROBE (Strengthening The Reporting of Observational Studies in Epidemiology) Checklist.

| Section and Item | Item No. | Recommendation | Reported on |
|---|---|---|---|
| **Title and Abstract** | 1 | (a) Indicate the study's design with a commonly used term in the title or the abstract. | The study design was specified in the abstract. Page 1. |
| | | (b) Provide in the abstract an informative and balanced summary of what was done and what was found. | Information on the methods and main results was provided in the abstract. Page 1. |
| **Introduction** | | | |
| Background/Rationale | 2 | Explain the scientific background and rationale for the investigation being reported. | The scientific background and interest of the study are explained in the introduction. Pages 1, 2. |
| Objectives | 3 | State specific objectives, including any prespecified hypotheses. | The objectives are stated in the last paragraph of the introduction. Page 2. |
| **Methods** | | | |
| Study Design | 4 | Present key elements of study design early in the paper. | The study design is presented in Methods (2.1). Page 2. |
| Setting | 5 | Describe the setting, locations, and relevant dates, including periods of recruitment, exposure, follow-up, and data collection. | The setting, locations and relevant dates are described in Methods (2.1). Page 2. Data collection is described in Methods (2.2.). Page 3. |
| Participants | 6 | Give the eligibility criteria, and the sources and methods of case ascertainment and control selection. Give the rationale for the choice of cases and controls. | The eligibility criteria, as well as the methods of case ascertainment are included in Methods (2.1). Page 2. |
| Variables | 7 | Clearly define all outcomes, exposures, predictors, potential confounders, and effect modifiers. Give diagnostic criteria, if applicable. | Variables are detailed in Methods (2.2). Page 3. A complete list of variables and their definitions are provided in Supplementary Material S2. |
| Data Sources/ Measurement | 8 | For each variable of interest, give sources of data and details of methods of assessment (measurement). Describe comparability of assessment methods if there is more than one group. | Data sources are explained in Methods (2.2). Page 3. Assessment methods were the same for every patient. |
| Bias | 9 | Describe any efforts to address potential sources of bias. | Selection bias: all (consecutive) cases who met the eligibility criteria were included. Information bias: standard and well-defined variables were used. Confusion bias: statistical analyses were performed adjusting by potential confounders. Page 3. |
| Study Size | 10 | Explain how the study size was arrived at. | All available cases were included, there was no size determination. |
| Quantitative Variables | 11 | Explain how quantitative variables were handled in the analyses. If applicable, describe which groupings were chosen and why. | Quantitative variables were presented as such (continuous except for CURB-65 score, which is discrete) and also categorized in the bivariate analysis; they were treated as quantitative in the multivariate analysis. |
| Statistical Methods | 12 | (a) Describe all statistical methods, including those used to control for confounding. | (a) Detailed in Methods (2.3). Page 3. |
| | | b) Describe any methods used to examine subgroups and interactions. | (b) We compared subjects with concurrent infection vs. those without (replicating the same multivariate analysis detailed in 2.3 in both subsets of patients) to explore the effect of treatment with azithromycin. |
| | | (c) Explain how missing data were addressed. | (c) Explained in Methods (2.2). Page 3. |
| | | (d) If applicable, explain how matching of cases and controls was addressed. | (d) Not applicable. |
| | | (e) Describe any sensitivity analyses. | (e) Not applicable. |
| **Results** | | | |

| | | | |
|---|---|---|---|
| Participants | 13 | (a) Report numbers of individuals at each stage of study–eg numbers potentially eligible, examined for eligibility, confirmed eligible, included in the study, completing follow-up, and analysed.<br>(b) Give reasons for non-participation at each stage.<br>(c) Consider use of a flow diagram. | (a) Explained in the first paragraph of Results. Page 4.<br>(b) Only one patient was excluded, the reason (age) is provided in Results. Page 4.<br>(c) Not useful in this study. |
| Participants | 14 | (a) Give characteristics of study participants (eg demographic, clinical, social) and information on exposures and potential confounders. | (a) Given in Table 1. Pages 5, 6. Distribution of age and sex is detailed in Figure 1. Page 4. Correlation matrices of comorbidities are presented in Figure 2. Page 7. |
| | | (b) Indicate the number of participants with missing data for each variable of interest. | (b) Included in Table 1. Pages 5, 6. |
| Outcome Data | 15 | Report numbers in each exposure category, or summary measures of exposure. | Reported in Table 1. Pages 5, 6. |
| Main Results | 16 | (a) Give unadjusted estimates and, if applicable, confounder-adjusted estimates and their precision (eg, 95% confidence interval). Make clear which confounders were adjusted for and why they were included.<br>(b) Report category boundaries when continuous variables were categorized.<br>(c) If relevant, consider translating estimates of relative risk into absolute risk for a meaningful time period. | (a) Unadjusted and adjusted estimates, along with their precision are presented in Table 2. Page 10.<br>(b) Reported in Table 1. Pages 5, 6.<br>(c) Not applicable. |
| Other Analyses | 17 | Report other analyses done–eg analyses of subgroups and interactions, and sensitivity analyses. | Other analyses (eg survival analysis) are reported in Results (3.1. and 3.2.). Pages 4-9. They are also shown in Figures 2, 3, and 4. Pages 7, 8 and 9, respectively. |
| | | **Discussion** | |
| Key Results | 18 | Summarize key results with reference to study objectives. | Summarized in Conclusions. Page 13. |
| Limitations | 19 | Discuss limitations of the study, taking into account sources of potential bias or imprecision. Discuss both direction and magnitude of any potential bias. | Included in Discussion (4.3.). Page 13. |
| Interpretation | 20 | Give a cautious overall interpretation of results considering objectives, limitations, multiplicity of analyses, results from similar studies, and other relevant evidence. | A comprehensive interpretation of the study results is provided throughout Discussion (4.1. and 4.2.). Pages 10-13. |
| Generalizability | 21 | Discuss the generalizability (external validity) of the study results. | Included in Discussion (4.3.). Page 13. |
| | | **Other information** | |
| Funding | 22 | Give the source of funding and the role of the funders for the present study and, if applicable, for the original study on which the present article is based. | Included in Funding. Page 13. |

**Table S2.** List of variables and their definitions.

| Variable | Definition |
|---|---|
| **Sociodemographic characteristics** | |
| Age | Age at the time of COVID-19 diagnosis, in years |
| Sex | Considered as binary: male or female |
| Country of birth | Patients were considered native if they were born in Spain and non-native if not |
| Place of residence | Living at home, nursing homes (part-time or full time) and institutions for people with disabilities (part-time or full-time) |
| Dependency in activities of daily living | Patients were considered dependent in activities of daily living if they required a caregiver (formal or informal) |
| **Chronic conditions** | |
| Arterial hypertension | History of arterial hypertension (essential or secondary) |
| Diabetes | History of type 1 or type 2 diabetes mellitus |
| Cardiovascular disease | History of coronary artery disease, chronic cardiac insufficiency, venous thromboembolism, cardiac arrhythmia, valvular heart disease or myocardiopathy |
| Chronic lung disease | History of non-allergic asthma, chronic obstructive pulmonary disease, interstitial lung disease, obstructive sleep apnea syndrome or cystic fibrosis |
| Chronic obstructive pulmonary disease | History of chronic obstructive pulmonary disease |
| Non-allergic asthma | History of intrinsic asthma, i.e., triggered by factors other than allergens |
| Chronic kidney disease | History of chronic kidney disease, defined by signs of kidney damage or by a glomerular filtration rate under 90 ml/min/1.73m$^2$ |
| Active cancer | Any solid or hematologic malignancy (excluding non-melanoma skin cancer) at the time of COVID-19 diagnosis |
| History of cancer in the previous five years | History of any solid or hematologic malignancy (excluding non-melanoma skin cancer) in the previous five years, in remission at the time of COVID-19 diagnosis |
| Autoimmune disease | History of any autoimmune disease (rheumatoid arthritis, inflammatory bowel disease, multiple sclerosis, psoriasis, celiac disease, systemic lupus erythematosus…) |
| Obesity | Body mass index of 30 and above at the time of COVID-19 diagnosis, in kg/m$^2$ |
| Tobacco smoking | Status of current tobacco smoker (any number of cigarettes) at the time of COVID-19 diagnosis |
| History of previous transplantation | History of any previous solid organ or hematopoietic stem-cell transplantation |
| HIV infection | History of confirmed infection by human immunodeficiency virus (HIV), regardless of the viral load and the CD4+ T-cell count |
| **Treatments received** | |
| Polymedication prior to admission | Patients were considered polymedicated if they usually received six or more different drugs prior to admission |
| Immunosuppressive therapy prior to admission | Patients were considered medically immunosuppressed if they usually received drugs responsible for high-level immunosuppression (≥20 mg/day of prednisone or equivalent for at least 14 consecutive days, ≥3 mg/kg/day of azathioprine, ≥1.5 mg/kg/day of mercaptopurine, chemotherapy, biologic therapies…) |
| Hydroxychloroquine | Administration of any dose of hydroxychloroquine to treat COVID-19 during hospital stay |
| Lopinavir-ritonavir | Administration of any dose of lopinavir-ritonavir to treat COVID-19 during hospital stay |
| Azithromycin | Administration of any dose of azithromycin to treat COVID-19 during hospital stay |
| Other antibiotics | Administration of any dose of any antibiotics other than azithromycin to treat COVID-19 during hospital stay |
| Tocilizumab | Administration of any dose of tocilizumab to treat COVID-19 during hospital stay |
| High-dose systemic corticosteroids | Administration of corticosteroid boluses or more than 40 mg/day of prednisone or equivalents (e.g., 6 mg/day of dexamethasone) for at least 7 consecutive days to treat COVID-19 during hospital stay |
| **Clinical data** | |
| Abnormal chest X-ray at admission | Abnormal radiographic findings attributable to COVID-19 (ground glass opacities, interstitial thickening, consolidations...) in the first chest X-ray performed within the first 5 days of hospitalisation |
| Ferritin levels upon admission | Serum ferritin levels in the first blood test performed within the first 5 days of hospitalisation |
| Acute distress respiratory syndrome | Acute distress respiratory syndrome during hospital stay, according to the 2012 Berlin definition: 1) onset over 7 days or less, 2) radiographic findings consistent with pulmonary oedema, 3) $PaO_2/FiO_2$ ratio <300 mm Hg receiving positive end-expiratory pressure (PEEP) or continuous positive airway pressure (CPAP) of at least 5 cm $H_2O$, and 4) not fully explained by cardiac failure or fluid overload |
| Concurrent infection | Coinfection or superinfection by any pathogen other than SARS-CoV-2 during hospital stay |
| Need for invasive mechanical ventilation | Need for invasive mechanical ventilation (IMV) was considered when patients were ventilated by orotracheal intubation or by tracheostomy tube |

| | |
|---|---|
| Need for non-invasive mechanical ventilation | Need for non-invasive mechanical ventilation (NIMV) was considered when patients were ventilated by continuous positive airway pressure (CPAP) or bilevel positive airway pressure (BiPAP) |
| Length of stay | Total length of hospital and intensive care unit (ICU) stay, considering the first hospital and ICU admission, respectively, as well as readmissions due to active SARS-CoV-2 infection (therefore excluding those due to COVID-19 sequelae), in days |
| CURB-65 Severity score upon admission | Result of the CURB-65 score applied upon hospital admission. It is a five point severity score, one point for each of: confusion, urea >7 mmol/l, respiratory rate ≥30/min, low systolic (<90 mm Hg) or diastolic (≤60 mm Hg) blood pressure and age ≥65 years |
| **Outcomes** | |
| Admission to the intensive care unit | Admission to the intensive care unit at any point during hospital stay |
| Death | Death due to any cause at any point during hospital stay |

**Table S3.** Cox regression model for in-hospital mortality among all admitted patients.

| Variables | Crude HR (95% CI) | *p* value [a] | Adjusted HR (95% CI) | *p* value [a] |
|---|---|---|---|---|
| **Age**, years | 1.08 (1.06-1.09) | <0.001 | 1.05 (1.03-1.07) | <0.001 |
| **Male sex** | 1.06 (0.78-1.44) | 0.696 | 1.25 (0.88-1.77) | 0.211 |
| **Active cancer** | 2.13 (1.31-3.49) | 0.002 | 2.54 (1.38-4.70) | 0.003 |
| **CURB-65 score upon admission** | 2.64 (2.24-3.11) | <0.001 | 2.03 (1.67-2.47) | <0.001 |
| **Azithromycin treatment** | 0.32 (0.23-0.44) | <0.001 | 0.49 (0.35-0.70) | <0.001 |
| **Non-invasive mechanical ventilation** | 0.39 (0.22-0.68) | <0.001 | 0.48 (0.25-0.91) | 0.024 |

CI, confidence interval; CURB-65, prognostic scale based on blood urea nitrogen, respiratory rate, blood pressure and age; HR, hazard ratio. [a] p-value of Wald's test. The concordance index of the model was 0.85.
